# Supplementary material for: Electrical activity controls area-specific expression of neuronal apoptosis in the mouse developing cerebral cortex
Source: eLife. 2017 Aug 21;6:e27696. doi: 10.7554/eLife.27696 (PMC5582867; doi:10.7554/eLife.27696)
Supplement: Figure 1—source data 1. — n = number of mice; sd = standard deviation; sem = standard error of mean. [file elife-27696-fig1-data1.docx]

Figure 1A. Quantification of aCasp3-positive cells/mm² in M1 and S1 regions of newborn mice. n=number of mice; sd= standard deviation; sem= standard error of mean.

|  | **P2** | **P3** | **P4** | **P5** | **P6** | **P7** | **P8** | **P9** | **P10** |
| --- | --- | --- | --- | --- | --- | --- | --- | --- | --- |
| **mean** | 5,030702 | 14,66816 | 27,20564 | 36,21732 | 34,30905 | 18,26712 | 11,48319 | 5,065816 | 4,710223 |
| **n** | 7 | 4 | 4 | 2 | 6 | 3 | 2 | 4 | 6 |
| **sd** | 4,121292 | 2,794654 | 7,702502 | 3,026339 | 29,53455 | 2,359112 | 6,371221 | 1,073357 | 1,881148 |
| **sem** | 1,557702 | 1,397327 | 3,851251 | 2,139945 | 12,05743 | 1,362034 | 4,505134 | 0,536679 | 0,767976 |
