# Supplementary material for: Electrical activity controls area-specific expression of neuronal apoptosis in the mouse developing cerebral cortex
Source: eLife. 2017 Aug 21;6:e27696. doi: 10.7554/eLife.27696 (PMC5582867; doi:10.7554/eLife.27696)
Supplement: Figure 3—source data 1. — n = number of slices analyzed; sd = standard deviation; sem = standard error of mean. [file elife-27696-fig3-data1.docx]

Figure 3A. Quantitative analysis of the density of aCasp3-positive cells in layers I-IV, V and VI of P5-7 mouse neocortex. n=number of slices analyzed; sd= standard deviation; sem= standard error of mean.

|  | **P5-7, layers I-IV** | | | |
| --- | --- | --- | --- | --- |
| **sectors** | **mean** | **n** | **sd** | **sem** |
| **a** | 86,64396 | 11 | 85,75496 | 25,85609 |
| **b** | 39,91802 | 11 | 41,14532 | 12,40578 |
| **c** | 16,51084 | 11 | 12,32096 | 3,71491 |
| **d** | 9,975823 | 11 | 5,808499 | 1,751328 |
| **e** | 11,92629 | 11 | 11,9564 | 3,604989 |
| **f** | 16,31054 | 10 | 7,18333 | 2,271568 |

|  | **P5-7, layer V** | | | |
| --- | --- | --- | --- | --- |
| **sectors** | **mean** | **n** | **sd** | **sem** |
| **a** | 12,02229 | 16 | 11,75921 | 2,939802 |
| **b** | 15,55395 | 16 | 15,38657 | 3,846643 |
| **c** | 26,59143 | 16 | 13,57231 | 3,393078 |
| **d** | 33,35255 | 16 | 19,90654 | 4,976634 |
| **e** | 12,10772 | 16 | 8,179223 | 2,044806 |
| **f** | 12,35917 | 15 | 9,451206 | 2,440291 |

|  | **P5-7, layer VI** | | | |
| --- | --- | --- | --- | --- |
| **sectors** | **mean** | **n** | **sd** | **sem** |
| **a** | 7,571126 | 5 | 4,504979 | 2,014688 |
| **b** | 5,163374 | 5 | 5,633418 | 2,519341 |
| **c** | 9,04175 | 5 | 6,378518 | 2,85256 |
| **d** | 7,920822 | 5 | 5,699448 | 2,548871 |
| **e** | 5,267202 | 5 | 6,897625 | 3,084712 |
| **f** | 6,136898 | 5 | 4,397361 | 1,96656 |
