# Supplementary material for: Electrical activity controls area-specific expression of neuronal apoptosis in the mouse developing cerebral cortex
Source: eLife. 2017 Aug 21;6:e27696. doi: 10.7554/eLife.27696 (PMC5582867; doi:10.7554/eLife.27696)
Supplement: Figure 3—source data 2. — n = number of slices analyzed; sd = standard deviation; sem = standard error of mean. [file elife-27696-fig3-data2.docx]

Figure 3B. Quantitative analysis of the density of TUNEL-positive cells in layers I-IV of P5-7 mice neocortex. n=number of slices analyzed; sd= standard deviation; sem= standard error of mean.

|  | **P5-7, layers I-IV** | | | | |  |  |  |  |  |
| --- | --- | --- | --- | --- | --- | --- | --- | --- | --- | --- |
| **sectors** | **mean** | **n** | **sd** | **sem** |  |  |  |  |  |  |
| **a** | 61,64998 | 6 | 37,02389 | 15,11494 |  |  |  |  |  |  |
| **b** | 45,98424 | 6 | 13,39061 | 5,466694 |  |  |  |  |  |  |
| **c** | 26,57732 | 6 | 7,888484 | 3,22046 |  |  |  |  |  |  |
| **d** | 15,11928 | 6 | 5,924911 | 2,418835 |  |  |  |  |  |  |
| **e** | 15,4146 | 6 | 9,513921 | 3,884042 |  |  |  |  |  |  |
| **f** | 18,71417 | 6 | 10,8977 | 4,448967 |  |  |  |  |  |  |
|  | | | | | | |  |  |  |  |
|  | | | | | | |  |  |  |  |
|  | | | | | | |  |  |  |  |
|  | | | | | | |  |  |  |  |
