# Supplementary material for: Electrical activity controls area-specific expression of neuronal apoptosis in the mouse developing cerebral cortex
Source: eLife. 2017 Aug 21;6:e27696. doi: 10.7554/eLife.27696 (PMC5582867; doi:10.7554/eLife.27696)
Supplement: Figure 3—source data 3. — n = number of slices analyzed; sd = standard deviation; sem = standard error of mean. [file elife-27696-fig3-data3.docx]

Figure 3C. Quantitative analysis of the density of apoptotic cells in layers I-IV, V and VI of P1-3 mouse neocortex. n=number of slices analyzed; sd= standard deviation; sem= standard error of mean.

| \|  \| **P1-3, layers I-IV** \| \| \| \| \| --- \| --- \| --- \| --- \| --- \| \| **sectors** \| **mean** \| **n** \| **sd** \| **sem** \| \| **a** \| 12,0177 \| 12 \| 11,24685 \| 3,246685 \| \| **b** \| 8,865866 \| 12 \| 11,00996 \| 3,178301 \| \| **c** \| 7,493504 \| 12 \| 8,372054 \| 2,416804 \| \| **d** \| 9,060582 \| 12 \| 8,569607 \| 2,473832 \| \| **e** \| 9,711147 \| 12 \| 11,76739 \| 3,396953 \| \| **f** \| 7,415105 \| 12 \| 10,12496 \| 2,922824 \| |  |  |  |  |
| --- | --- | --- | --- | --- | --- | --- | --- | --- | --- | --- | --- | --- | --- | --- | --- | --- | --- | --- | --- | --- | --- | --- | --- | --- | --- | --- | --- | --- | --- | --- | --- | --- | --- | --- | --- | --- | --- | --- | --- | --- | --- | --- | --- | --- |
|  |  |  |  |  |
|  |  |  |  |  |
| \|  \| **P1-3, layer V** \| \| \| \| \| --- \| --- \| --- \| --- \| --- \| \| **sectors** \| **mean** \| **n** \| **sd** \| **sem** \| \| **a** \| 16,51036 \| 12 \| 13,3861 \| 3,864233 \| \| **b** \| 10,66181 \| 12 \| 11,36085 \| 3,279594 \| \| **c** \| 10,76256 \| 12 \| 9,131187 \| 2,635947 \| \| **d** \| 9,266871 \| 12 \| 11,55017 \| 3,334246 \| \| **e** \| 15,89826 \| 12 \| 13,33446 \| 3,849328 \| \| **f** \| 11,60493 \| 12 \| 14,03655 \| 4,052002 \| |  |  |  |  |
|  |  |  |  |  |
|  |  |  |  |  |
| \|  \| **P1-3, layer VI** \| \| \| \| \| --- \| --- \| --- \| --- \| --- \| \| **sectors** \| **mean** \| **n** \| **sd** \| **sem** \| \| **a** \| 5,316711 \| 5 \| 4,953786 \| 2,2154 \| \| **b** \| 7,764674 \| 5 \| 9,213701 \| 4,120492 \| \| **c** \| 4,810178 \| 5 \| 7,030871 \| 3,144301 \| \| **d** \| 6,226521 \| 5 \| 6,530019 \| 2,920313 \| \| **e** \| 15,06111 \| 5 \| 24,39271 \| 10,90875 \| \| **f** \| 11,7547 \| 5 \| 12,18071 \| 5,447381 \| |  |  |  |  |
