# Supplementary material for: Electrical activity controls area-specific expression of neuronal apoptosis in the mouse developing cerebral cortex
Source: eLife. 2017 Aug 21;6:e27696. doi: 10.7554/eLife.27696 (PMC5582867; doi:10.7554/eLife.27696)
Supplement: Figure 3—source data 4. — n=number of slices analyzed; sd= standard deviation; sem= standard error of mean. [file elife-27696-fig3-data4.docx]

Figure 3D. Quantitative analysis of the density of apoptotic cells in layers I-IV, V and VI of P9-11 mouse neocortex. n=number of slices analyzed; sd= standard deviation; sem= standard error of mean.

|  | | | | | |  |  |  |  |
| --- | --- | --- | --- | --- | --- | --- | --- | --- | --- |
|  | | | | | |  |  |  |  |
|  | **P9-11, layers I-IV** | | | |  |  |  |  |  |
| **sectors** | **mean** | **n** | **sd** | **sem** |  |  |  |  |  |
| **a** | 7,983958 | 12 | 7,300204 | 2,107387 |  |  |  |  |  |
| **b** | 2,167163 | 12 | 2,970634 | 0,857548 |  |  |  |  |  |
| **c** | 4,122793 | 12 | 4,306985 | 1,243319 |  |  |  |  |  |
| **d** | 4,736647 | 12 | 4,428543 | 1,27841 |  |  |  |  |  |
| **e** | 4,397112 | 12 | 3,171401 | 0,915505 |  |  |  |  |  |
| **f** | 3,497746 | 12 | 3,029039 | 0,874408 |  |  |  |  |  |

|  | **P9-11, layers I-IV** | | | |
| --- | --- | --- | --- | --- |
| **sectors** | **mean** | **n** | **sd** | **sem** |
| **a** | 8,161824 | 10 | 8,200824 | 2,593328 |
| **b** | 15,40261 | 10 | 12,26235 | 3,877695 |
| **c** | 13,25564 | 10 | 11,03067 | 3,488204 |
| **d** | 9,963119 | 10 | 13,75118 | 4,348506 |
| **e** | 10,29083 | 10 | 13,03966 | 4,123502 |
| **f** | 4,620709 | 9 | 6,289314 | 2,096438 |

|  | **P9-11, layers I-VI** | | | |
| --- | --- | --- | --- | --- |
| **sectors** | **mean** | **n** | **sd** | **sem** |
| **a** | 4,991142 | 6 | 4,973181 | 2,030293 |
| **b** | 0,768049 | 6 | 1,881329 | 0,768049 |
| **c** | 5,861915 | 6 | 3,63065 | 1,482206 |
| **d** | 0 | 6 | 0 | 0 |
| **e** | 3,716503 | 6 | 4,995178 | 2,039273 |
| **f** | 1,338688 | 6 | 3,279103 | 1,338688 |
