# Supplementary material for: Electrical activity controls area-specific expression of neuronal apoptosis in the mouse developing cerebral cortex
Source: eLife. 2017 Aug 21;6:e27696. doi: 10.7554/eLife.27696 (PMC5582867; doi:10.7554/eLife.27696)
Supplement: Figure 5—source data 1. — n=number of slices analyzed; sd= standard deviation; sem= standard error of mean. [file elife-27696-fig5-data1.docx]

Figure 5A3. Quantitative analysis of the density of aCasp3-positive cells in layers I-IV of acute slices from P5-7 or adult mouse neocortex perfused with control or 4-AP/no Mg^2+^ ACSF. n=number of slices analyzed; sd= standard deviation; sem= standard error of mean.

|  | **aCasp3-positive cells/mm²** | | | | | | | |
| --- | --- | --- | --- | --- | --- | --- | --- | --- |
|  | **P5-7, ctrl** | | | | **P5-7, 4-AP/no Mg^2+^** | | | |
| **sectors** | **mean** | **n** | **sd** | **sem** | **mean** | **n** | **sd** | **sem** |
| **a** | 272,7492 | 7 | 179,1048 | 67,69526 | 136,1637 | 7 | 106,2172 | 40,14633 |
| **b** | 233,9046 | 7 | 65,76891 | 24,85831 | 72,32547 | 7 | 61,00186 | 23,05653 |
| **c** | 97,9816 | 7 | 44,05401 | 16,65085 | 30,38828 | 7 | 19,52284 | 7,37894 |
| **d** | 47,98968 | 7 | 22,44119 | 8,481974 | 37,4589 | 7 | 25,62243 | 9,68437 |
| **e** | 56,4278 | 7 | 50,47485 | 19,0777 | 31,37906 | 7 | 25,22702 | 9,534916 |
| **f** | 86,96108 | 7 | 42,47628 | 16,05452 | 47,78815 | 7 | 45,60338 | 17,23646 |

|  | **aCasp3-positive cells/mm²** | | | | | | | |
| --- | --- | --- | --- | --- | --- | --- | --- | --- |
|  | **adult, ctrl** | | | | **adult, 4-AP/no Mg^2+^** | | | |
| **sectors** | **mean** | **n** | **sd** | **sem** | **mean** | **n** | **sd** | **sem** |
| **a** | 7,324938 | 6 | 7,768101 | 3,171314 | 6,608511 | 6 | 5,143528 | 2,099837 |
| **b** | 10,03345 | 6 | 9,51619 | 3,884968 | 6,924013 | 6 | 4,050262 | 1,653513 |
| **c** | 10,45691 | 6 | 7,642369 | 3,119984 | 11,4995 | 6 | 8,292695 | 3,385478 |
| **d** | 9,276644 | 6 | 2,936414 | 1,198786 | 13,39106 | 6 | 14,01029 | 5,719675 |
| **e** | 4,39655 | 6 | 4,600127 | 1,877994 | 7,122176 | 6 | 3,832173 | 1,564478 |
| **f** | 2,412149 | 6 | 3,742233 | 1,52776 | 9,463963 | 6 | 7,149932 | 2,918947 |
