# Supplementary material for: Electrical activity controls area-specific expression of neuronal apoptosis in the mouse developing cerebral cortex
Source: eLife. 2017 Aug 21;6:e27696. doi: 10.7554/eLife.27696 (PMC5582867; doi:10.7554/eLife.27696)
Supplement: Figure 5—source data 2. — n=number of slices analyzed; sd= standard deviation; sem= standard error of mean. [file elife-27696-fig5-data2.docx]

Figure 5B3. Quantitative analysis of the density of aCasp3-positive cells in layers I-IV of acute slices from P5-7 mouse neocortex perfused with control or gabazine/no Mg^2+^ ACSF. n=number of slices analyzed; sd= standard deviation; sem= standard error of mean.

|  | **aCasp3-positive cells/mm²** | | | | | | | |
| --- | --- | --- | --- | --- | --- | --- | --- | --- |
|  | **P5-7, ctrl** | | | | **P5-7, Gbz/no Mg^2+^** | | | |
| **sectors** | **mean** | **n** | **sd** | **sem** | **mean** | **n** | **sd** | **sem** |
| **a** | 264,8253 | 6 | 131,0399 | 53,49684 | 143,1102 | 7 | 144,6668 | 54,67891 |
| **b** | 193,0759 | 6 | 146,2254 | 59,69627 | 107,3223 | 7 | 82,98901 | 31,3669 |
| **c** | 145,9319 | 6 | 94,72881 | 38,67287 | 60,76095 | 7 | 44,00558 | 16,63255 |
| **d** | 96,03336 | 6 | 79,34262 | 32,39149 | 59,17255 | 7 | 49,10046 | 18,55823 |
| **e** | 118,3447 | 6 | 58,29826 | 23,80017 | 57,72665 | 7 | 42,19586 | 15,94853 |
| **f** | 122,3352 | 6 | 119,3036 | 48,70548 | 78,3138 | 7 | 45,80418 | 17,31235 |
