# Supplementary material for: Electrical activity controls area-specific expression of neuronal apoptosis in the mouse developing cerebral cortex
Source: eLife. 2017 Aug 21;6:e27696. doi: 10.7554/eLife.27696 (PMC5582867; doi:10.7554/eLife.27696)
Supplement: Figure 6—source data 1. — n=number of hemispheres analyzed; sd= standard deviation; sem= standard error of mean. [file elife-27696-fig6-data1.docx]

Figure 6C. Quantitative analysis of the density of aCasp3-positive cells in layers I-IV of acute slices from P5-7 mouse neocortex with and without replay of S1 and M1 *in vivo* activity. n=number of hemispheres analyzed; sd= standard deviation; sem= standard error of mean.

|  | no stimulation | | | | replay of *in vivo* activity | | | |
| --- | --- | --- | --- | --- | --- | --- | --- | --- |
| sectors | mean | n | sd | sem | mean | n | sd | Sem |
| a | 203,8255 | 12 | 116,3825 | 33,59673 | 144,8591 | 12 | 61,13758 | 17,6489 |
| b | 157,0342 | 12 | 106,8775 | 30,85289 | 100,4169 | 12 | 42,312 | 12,21442 |
| c | 100,0876 | 12 | 51,7519 | 14,93949 | 52,86525 | 12 | 33,37016 | 9,633134 |
| d | 63,12535 | 12 | 39,90547 | 11,51972 | 45,93603 | 12 | 26,10628 | 7,536233 |
| e | 62,61233 | 12 | 44,45738 | 12,83374 | 47,62906 | 12 | 34,27737 | 9,895024 |
| f | 105,0413 | 11 | 64,73683 | 19,51889 | 53,72665 | 12 | 39,93433 | 11,52805 |
