# Supplementary material for: Electrical activity controls area-specific expression of neuronal apoptosis in the mouse developing cerebral cortex
Source: eLife. 2017 Aug 21;6:e27696. doi: 10.7554/eLife.27696 (PMC5582867; doi:10.7554/eLife.27696)
Supplement: Figure 6—source data 2. — n=number of slices analyzed; sd= standard deviation; sem= standard error of mean. [file elife-27696-fig6-data2.docx]

Figure 6D. Quantification of decrease in the number of aCasp3-positive neurons following electrical stimulation (related to the unstimulated hemisphere). n=number of slices analyzed; sd= standard deviation; sem= standard error of mean.

|  | replay of M1 *in vivo* activity | | | | replay of S1 *in vivo* activity | | | |
| --- | --- | --- | --- | --- | --- | --- | --- | --- |
| sectors | mean | n | sd | sem | mean | n | sd | sem |
| a | -21,5461 | 6 | 16,71583 | 6,824207 | -97,8232 | 6 | 139,0905 | 56,78346 |
| b | -19,5224 | 6 | 19,79567 | 8,081547 | -57,6908 | 6 | 48,88702 | 19,95804 |
| c | -24,2867 | 6 | 51,21176 | 20,90711 | -46,0387 | 6 | 25,67227 | 10,48066 |
| d | -7,66606 | 6 | 55,80343 | 22,78165 | -15,0473 | 6 | 80,60309 | 32,90608 |
| e | -6,03621 | 6 | 45,3889 | 18,52994 | -64,1911 | 6 | 84,25517 | 34,39703 |
| f | -19,8038 | 6 | 70,26127 | 28,68405 | -28,1461 | 5 | 66,38619 | 29,68881 |
