# Supplementary material for: Electrical activity controls area-specific expression of neuronal apoptosis in the mouse developing cerebral cortex
Source: eLife. 2017 Aug 21;6:e27696. doi: 10.7554/eLife.27696 (PMC5582867; doi:10.7554/eLife.27696)
Supplement: Figure 7—source data 1. — n=number of slices analyzed; sd= standard deviation; sem= standard error of mean. [file elife-27696-fig7-data1.docx]

Figure 7A. Quantitative analysis of the density of aCasp3-positive cells in M1 and S1 areas of saline- or kainate-injected P5-7 mice. n=number of slices analyzed; sd= standard deviation; sem= standard error of mean.

|  | **Saline-injected mice** | | **Kainate-injected mice** | |
| --- | --- | --- | --- | --- |
|  | **M1** | **S1** | **M1** | **S1** |
| **mean** | 52,03408643 | 14,089402 | 30,25188076 | 9,282273941 |
| **n** | 14 | 14 | 17 | 17 |
| **sd** | 22,45632776 | 9,314332834 | 25,39972972 | 6,47589148 |
| **sem** | 6,001706046 | 2,489360161 | 6,160339323 | 1,570634388 |
