# Supplementary material for: Electrical activity controls area-specific expression of neuronal apoptosis in the mouse developing cerebral cortex
Source: eLife. 2017 Aug 21;6:e27696. doi: 10.7554/eLife.27696 (PMC5582867; doi:10.7554/eLife.27696)
Supplement: Figure 7—source data 2. — n=number of slices analyzed; sd= standard deviation; sem= standard error of mean. [file elife-27696-fig7-data2.docx]

Figure 7B. Quantitative analysis of the density of aCasp3-positive cells in S1 area of P5-7 mice. n=number of slices analyzed; sd= standard deviation; sem= standard error of mean.

|  | **Left hemisphere** | **Right hemisphere** |
| --- | --- | --- |
| **mean** | 18,8747885 | 14,1687657 |
| **n** | 20 | 20 |
| **sd** | 7,994991185 | 6,100204629 |
| **sem** | 1,787734377 | 1,364047223 |
