# Supplementary material for: Electrical activity controls area-specific expression of neuronal apoptosis in the mouse developing cerebral cortex
Source: eLife. 2017 Aug 21;6:e27696. doi: 10.7554/eLife.27696 (PMC5582867; doi:10.7554/eLife.27696)
Supplement: Transparent reporting form [file elife-27696-transrepform.docx]

***eLife’s* transparent reporting form**

**Sample-size estimation**

Based on previous results of our laboratory, a size of 3 to 10 samples was used. Sample size limitations for the statistical tests applied (t-test and ANOVA) were taken in consideration.

**Replicates**

Information can be found in material and method part (lines 624-625): “Experimental groups consisted of at least 5 biological replicates gathered from minimum 3 independent experiments”.

Ex: line 213, recording of 6 slices from at least 3 recording days.

**Statistical reporting**

lines 100; 111-113; 124-126; 129-130; 131-133; 137-138; 143-144; 145-147; 173-177; 181-184; 203-206; 212-213; 216-217; 222-223; 229; 232-233; 239-242; 252-253; 256-257; 278-281; 292-298; 312-314.

**Additional data files (“source data”)**

Figures 1, 3, 4, 5, 6, 7
